# 1,2-Bis(4-ethynylphen­yl)disulfane

**DOI:** 10.1107/S1600536810004721

**Published:** 2010-02-13

**Authors:** Qi Xiao, Rui Liu, Yu-Hao Li, Hong-Bin Chen, Hong-Jun Zhu

**Affiliations:** a Department of Applied Chemistry, College of Science, Nanjing University of Technology, Nanjing 210009, People’s Republic of China

## Abstract

In the title compound, C_16_H_10_S_2_, the S atoms are almost coplanar with the benzene rings to which they are bonded [deviations of 0.092 (1) and 0.022 (1) Å from their respective ring planes]. The benzene rings enclose a dihedral angle of 79.17 (3)°. An intra­molecular C—H⋯S hydrogen bond results in the formation of a five-membered ring. In the crystal structure, mol­ecules are stacked parallel to the *a* axis direction. π–π inter­actions between benzene rings are present, with a face-to-face stacking distance of 3.622 (10) Å.

## Related literature

For bond-length data, see: Allen *et al.* (1987 ▶). For the synthetic procedure, see Yonezawa *et al.* (2008 ▶).
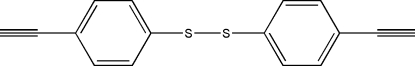


## Experimental

### 

#### Crystal data

C_16_H_10_S_2_
*M* *_r_* = 266.36
Triclinic, 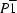

*a* = 5.981 (1) Å
*b* = 8.881 (2) Å
*c* = 13.269 (3) Å
α = 94.92 (3)°
β = 99.29 (3)°
γ = 104.45 (3)°
*V* = 667.7 (2) Å^3^
*Z* = 2
Mo *K*α radiation
μ = 0.38 mm^−1^
*T* = 293 K
0.40 × 0.40 × 0.30 mm

#### Data collection

Enraf–Nonius CAD-4 diffractometer
Absorption correction: ψ scan (North *et al.*, 1968 ▶) *T*_min_ = 0.864, *T*_max_ = 0.896
2887 measured reflections
2620 independent reflections
1969 reflections with *I* > 2σ(*I*)
*R*_int_ = 0.033
3 standard reflections every 200 reflections  intensity decay: none

#### Refinement

*R*[*F* ^2^ > 2σ(*F* ^2^)] = 0.060
*wR*(*F* ^2^) = 0.194
*S* = 1.01
2620 reflections
169 parameters
2 restraints
H atoms treated by a mixture of independent and constrained refinement
Δρ_max_ = 0.50 e Å^−3^
Δρ_min_ = −0.40 e Å^−3^

### 

Data collection: *CAD-4 Software* (Enraf–Nonius, 1985 ▶); cell refinement: *CAD-4 Software*; data reduction: *XCAD4* (Harms & Wocadlo,1995 ▶); program(s) used to solve structure: *SHELXS97* (Sheldrick, 2008 ▶); program(s) used to refine structure: *SHELXL97* (Sheldrick, 2008 ▶); molecular graphics: *SHELXTL* (Sheldrick, 2008 ▶); software used to prepare material for publication: *SHELXTL*.

## Supplementary Material

Crystal structure: contains datablocks I, XQ. DOI: 10.1107/S1600536810004721/im2180sup1.cif

Structure factors: contains datablocks I. DOI: 10.1107/S1600536810004721/im2180Isup2.hkl

Additional supplementary materials:  crystallographic information; 3D view; checkCIF report

## Figures and Tables

**Table 1:** Hydrogen-bond geometry (Å, °)

*D*—H⋯*A*	*D*—H	H⋯*A*	*D*⋯*A*	*D*—H⋯*A*
C13—H13*A*⋯S1	0.93	2.71	3.216 (5)	115

**Fig. 1.**
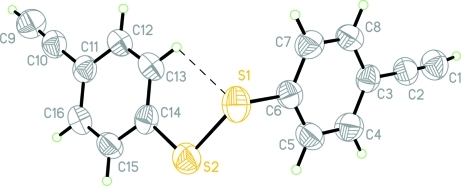
Molecular structure of (I), showing the atom-numbering scheme. Displacement ellipsoids are drawn at the 50% probability level.

**Fig. 2.**
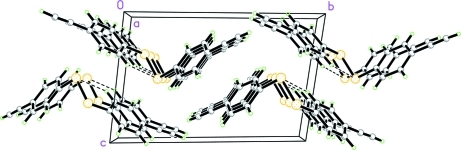
Packing diagram of (I). Hydrogen bonds are shown as dashed lines.

## supplementary crystallographic information

### Comment

The title compound, (I), is a kind of aromatic acetylide organic intermediate which can be used for many fields such as molecular electronic materials, organometallic chemistry *etc.* (Yonezawa *et al.*, 2008). We herein report its crystal structure.

In the molecule of (I), (Fig.1), the bond lengths and angles are within normal ranges (Allen *et al.*, 1987). S atoms are situated in the same plane as the benzene rings they are bonded to. Rings A (C3—C8) and B (C11—C16) are, of course, planar and they enclose a dihedral angle of 79.17 (3) °. An intramolecular C—H···S hydrogen bond (Table 1) results in the formation of a five-membered ring C (S1/S2/C14/C13/H13A). The distance between atoms S2 and H5A is 2.91 Å, which is significantly longer than the hydrogen bond between atoms S1 and H13A.

As can be seen from the packing diagram, (Fig. 2), the molecules are stacked along the *a* axis. There are also the π-π interactions of benzene rings with a face-to-face stacking distance of 3.622 Å.

### Experimental

The title compound, (I) was prepared by a literature method (Yonezawa *et al.*, 2008). Crystals suitable for X-ray analysis were obtained by dissolving (I) (0.5 g) in hexane (20 ml) and evaporating the solvent slowly at room temperature for about 7 d.

### Refinement

H atoms were positioned geometrically, with C—H = 0.93 Å for aromatic H and 0.95 Å for acetylide H. In the refinement all hydrogens were constrained to ride on their parent atoms, with *U*_iso_(H) = *xU*_eq_(C), with *x* = 1.2 for aromatic H, and *x* = 1.5 for other H.

### Crystal data

**Table:**

C_16_H_10_S_2_	*Z* = 2
*M**_r_* = 266.36	*F*(000) = 276
Triclinic, *P*1	*D*_x_ = 1.325 Mg m^−^^3^
Hall symbol: -P 1	Melting point: 391 K
*a* = 5.981 (1) Å	Mo *K*α radiation, λ = 0.71073 Å
*b* = 8.881 (2) Å	Cell parameters from 25 reflections
*c* = 13.269 (3) Å	θ = 10–13°
α = 94.92 (3)°	µ = 0.38 mm^−^^1^
β = 99.29 (3)°	*T* = 293 K
γ = 104.45 (3)°	Block, colourless
*V* = 667.7 (2) Å^3^	0.40 × 0.40 × 0.30 mm

### Data collection

**Table:**

Enraf–Nonius CAD-4 diffractometer	1969 reflections with *I* > 2σ(*I*)
Radiation source: fine-focus sealed tube	*R*_int_ = 0.033
graphite	θ_max_ = 26.0°, θ_min_ = 1.6°
ω/2θ scans	*h* = −7→7
Absorption correction: ψ scan (North *et al.*, 1968)	*k* = −10→10
*T*_min_ = 0.864, *T*_max_ = 0.896	*l* = 0→16
2887 measured reflections	3 standard reflections every 200 reflections
2620 independent reflections	intensity decay: none

### Refinement

**Table:**

Refinement on *F*^2^	Primary atom site location: structure-invariant direct methods
Least-squares matrix: full	Secondary atom site location: difference Fourier map
*R*[*F*^2^ > 2σ(*F*^2^)] = 0.060	Hydrogen site location: inferred from neighbouring sites
*wR*(*F*^2^) = 0.194	H atoms treated by a mixture of independent and constrained refinement
*S* = 1.01	*w* = 1/[σ^2^(*F*_o_^2^) + (0.060*P*)^2^ + 2.*P*] where *P* = (*F*_o_^2^ + 2*F*_c_^2^)/3
2620 reflections	(Δ/σ)_max_ < 0.001
169 parameters	Δρ_max_ = 0.50 e Å^−^^3^
2 restraints	Δρ_min_ = −0.40 e Å^−^^3^

### Special details

**Table:**

Geometry. All esds (except the esd in the dihedral angle between two l.s. planes) are estimated using the full covariance matrix. The cell esds are taken into account individually in the estimation of esds in distances, angles and torsion angles; correlations between esds in cell parameters are only used when they are defined by crystal symmetry. An approximate (isotropic) treatment of cell esds is used for estimating esds involving l.s. planes.
Refinement. Refinement of *F*^2^ against ALL reflections. The weighted *R*-factor wR and goodness of fit *S* are based on *F*^2^, conventional *R*-factors *R* are based on *F*, with *F* set to zero for negative *F*^2^. The threshold expression of *F*^2^ > σ(*F*^2^) is used only for calculating *R*-factors(gt) etc. and is not relevant to the choice of reflections for refinement. *R*-factors based on *F*^2^ are statistically about twice as large as those based on *F*, and *R*- factors based on ALL data will be even larger.

### Fractional atomic coordinates and isotropic or equivalent isotropic displacement parameters (Å^2^)

**Table:**

	*x*	*y*	*z*	*U*_iso_*/*U*_eq_	
S1	0.0903 (2)	0.50232 (15)	0.20304 (9)	0.0594 (4)	
C1	0.8307 (10)	0.2056 (7)	0.5708 (4)	0.0674 (13)	
S2	−0.1535 (2)	0.31363 (17)	0.11850 (10)	0.0619 (4)	
C2	0.7075 (8)	0.2523 (6)	0.5110 (4)	0.0561 (11)	
C3	0.5594 (8)	0.3096 (5)	0.4359 (3)	0.0479 (10)	
C4	0.3177 (9)	0.2406 (6)	0.4127 (4)	0.0606 (12)	
H4A	0.2522	0.1573	0.4461	0.073*	
C5	0.1745 (8)	0.2953 (6)	0.3402 (4)	0.0571 (11)	
H5A	0.0137	0.2475	0.3242	0.069*	
C6	0.2705 (8)	0.4209 (5)	0.2916 (3)	0.0476 (10)	
C7	0.5073 (8)	0.4891 (6)	0.3143 (4)	0.0575 (11)	
H7A	0.5722	0.5718	0.2803	0.069*	
C8	0.6516 (8)	0.4362 (6)	0.3876 (4)	0.0578 (11)	
H8A	0.8116	0.4863	0.4042	0.069*	
C9	0.3291 (10)	0.0564 (7)	−0.3034 (4)	0.0686 (14)	
C10	0.2502 (8)	0.0917 (6)	−0.2347 (4)	0.0561 (11)	
C11	0.1571 (8)	0.1440 (5)	−0.1475 (3)	0.0503 (10)	
C12	0.2959 (8)	0.2639 (5)	−0.0736 (3)	0.0522 (10)	
H12A	0.4508	0.3089	−0.0791	0.063*	
C13	0.2091 (8)	0.3179 (6)	0.0079 (4)	0.0567 (11)	
H13A	0.3057	0.3986	0.0570	0.068*	
C14	−0.0207 (8)	0.2532 (5)	0.0175 (3)	0.0490 (10)	
C15	−0.1597 (8)	0.1311 (5)	−0.0558 (4)	0.0542 (11)	
H15A	−0.3133	0.0845	−0.0493	0.065*	
C16	−0.0744 (8)	0.0782 (6)	−0.1376 (3)	0.0542 (11)	
H16A	−0.1714	−0.0022	−0.1869	0.065*	
H9	0.385 (8)	0.019 (5)	−0.362 (2)	0.065*	
H1	0.947 (6)	0.176 (6)	0.617 (3)	0.065*	

### Atomic displacement parameters (Å^2^)

**Table:**

	*U*^11^	*U*^22^	*U*^33^	*U*^12^	*U*^13^	*U*^23^
S1	0.0706 (8)	0.0593 (7)	0.0513 (7)	0.0280 (6)	0.0044 (5)	0.0062 (5)
C1	0.069 (3)	0.071 (3)	0.062 (3)	0.021 (3)	0.006 (3)	0.012 (3)
S2	0.0527 (7)	0.0807 (9)	0.0534 (7)	0.0229 (6)	0.0062 (5)	0.0069 (6)
C2	0.055 (3)	0.060 (3)	0.049 (3)	0.014 (2)	0.004 (2)	0.002 (2)
C3	0.051 (2)	0.055 (2)	0.042 (2)	0.023 (2)	0.0102 (18)	0.0059 (18)
C4	0.059 (3)	0.064 (3)	0.060 (3)	0.017 (2)	0.010 (2)	0.018 (2)
C5	0.044 (2)	0.062 (3)	0.059 (3)	0.011 (2)	0.001 (2)	0.006 (2)
C6	0.050 (2)	0.045 (2)	0.046 (2)	0.0144 (18)	0.0069 (18)	0.0012 (18)
C7	0.059 (3)	0.058 (3)	0.057 (3)	0.013 (2)	0.013 (2)	0.017 (2)
C8	0.047 (2)	0.063 (3)	0.057 (3)	0.006 (2)	0.004 (2)	0.010 (2)
C9	0.072 (3)	0.070 (3)	0.068 (3)	0.024 (3)	0.021 (3)	0.005 (3)
C10	0.059 (3)	0.056 (3)	0.053 (3)	0.017 (2)	0.004 (2)	0.009 (2)
C11	0.053 (3)	0.053 (2)	0.049 (2)	0.021 (2)	0.0062 (19)	0.0157 (19)
C12	0.046 (2)	0.058 (3)	0.050 (2)	0.014 (2)	0.0022 (19)	0.007 (2)
C13	0.043 (2)	0.067 (3)	0.053 (3)	0.010 (2)	−0.0027 (19)	0.005 (2)
C14	0.057 (3)	0.054 (2)	0.040 (2)	0.023 (2)	0.0075 (18)	0.0115 (18)
C15	0.040 (2)	0.060 (3)	0.057 (3)	0.010 (2)	−0.0013 (19)	0.010 (2)
C16	0.052 (3)	0.058 (3)	0.048 (2)	0.016 (2)	−0.0040 (19)	0.001 (2)

### Geometric parameters (Å, °)

**Table:**

S1—C6	1.786 (4)	C8—H8A	0.9300
S1—S2	2.030 (2)	C9—C10	1.147 (7)
C1—C2	1.169 (7)	C9—H9	0.959 (10)
C1—H1	0.956 (10)	C10—C11	1.452 (7)
S2—C14	1.774 (4)	C11—C12	1.379 (6)
C2—C3	1.436 (6)	C11—C16	1.394 (6)
C3—C8	1.382 (6)	C12—C13	1.374 (6)
C3—C4	1.393 (6)	C12—H12A	0.9300
C4—C5	1.384 (6)	C13—C14	1.382 (6)
C4—H4A	0.9300	C13—H13A	0.9300
C5—C6	1.383 (6)	C14—C15	1.386 (6)
C5—H5A	0.9300	C15—C16	1.368 (7)
C6—C7	1.366 (6)	C15—H15A	0.9300
C7—C8	1.386 (6)	C16—H16A	0.9300
C7—H7A	0.9300		
			
C6—S1—S2	104.69 (15)	C7—C8—H8A	119.8
C2—C1—H1	173 (3)	C10—C9—H9	175 (3)
C14—S2—S1	105.55 (17)	C9—C10—C11	177.3 (6)
C1—C2—C3	178.8 (5)	C12—C11—C16	118.5 (4)
C8—C3—C4	118.7 (4)	C12—C11—C10	120.2 (4)
C8—C3—C2	121.0 (4)	C16—C11—C10	121.4 (4)
C4—C3—C2	120.3 (4)	C13—C12—C11	121.0 (4)
C5—C4—C3	120.4 (4)	C13—C12—H12A	119.5
C5—C4—H4A	119.8	C11—C12—H12A	119.5
C3—C4—H4A	119.8	C12—C13—C14	120.6 (4)
C6—C5—C4	120.0 (4)	C12—C13—H13A	119.7
C6—C5—H5A	120.0	C14—C13—H13A	119.7
C4—C5—H5A	120.0	C13—C14—C15	118.5 (4)
C7—C6—C5	119.7 (4)	C13—C14—S2	124.8 (4)
C7—C6—S1	118.7 (3)	C15—C14—S2	116.7 (4)
C5—C6—S1	121.5 (3)	C16—C15—C14	121.0 (4)
C6—C7—C8	120.6 (4)	C16—C15—H15A	119.5
C6—C7—H7A	119.7	C14—C15—H15A	119.5
C8—C7—H7A	119.7	C15—C16—C11	120.4 (4)
C3—C8—C7	120.5 (4)	C15—C16—H16A	119.8
C3—C8—H8A	119.8	C11—C16—H16A	119.8

### Hydrogen-bond geometry (Å, °)

**Table:**

*D*—H···*A*	*D*—H	H···*A*	*D*···*A*	*D*—H···*A*
C13—H13A···S1	0.93	2.71	3.216 (5)	115
